# Biocompatibility of Hydraulic Calcium Silicate-Based Cement MTA Flow^TM^ on Human Dental Pulp Stem Cells In Vitro

**DOI:** 10.3390/jfb16070252

**Published:** 2025-07-07

**Authors:** Paulius Tušas, Josette Camilleri, Milda Alksnė, Egidijus Šimoliūnas, Saulius Drukteinis, Eglė Marija Urbonė, Virginija Bukelskienė, Vygandas Rutkūnas, Vytautė Pečiulienė

**Affiliations:** 1 Institute of Dentistry, Faculty of Medicine, Vilnius University, Zalgirio 115, LT-08217 Vilnius, Lithuania; saulius.drukteinis@mf.vu.lt (S.D.); egle.urbone@mf.vu.lt (E.M.U.); vygandas.rutkunas@mf.vu.lt (V.R.); vytaute.peciuliene@mf.vu.lt (V.P.); 2 School of Dentistry, Institute of Clinical Sciences, College of Medical and Dental Sciences, University of Birmingham, 5 Mill Pool Way, Birmingham B5 7EG, UK; j.camilleri@bham.ac.uk; 3 Department of Biological Models, Life Science Center, Institute of Biochemistry, Vilnius University, Sauletekio al. 7, LT-10257 Vilnius, Lithuania; milda.peciukaityte@gf.vu.lt (M.A.); egidijus.simoliunas@gmc.vu.lt (E.Š.); virginija.bukelskiene@gmc.vu.lt (V.B.)

**Keywords:** biocompatibility, hydraulic calcium silicate-based cement, human dental pulp stem cells, cell proliferation, apoptosis assay

## Abstract

Aim: hydraulic calcium silicate-based cements (HCSCs) are widely used in endodontics for vital pulp therapy and other clinical procedures due to their favorable physicochemical and biological properties. This study evaluates the biological properties of two HCSCs—MTA Flow™ and MTA Flow™ White (in a 3:2 liquid-to-powder ratio, thick consistency)—on human dental pulp stem cells (hDPSCs). Methodology: hDPSCs were exposed to leachates from MTA Flow™, MTA Flow™ White, and ProRoot^®^ MTA. pH changes, cytotoxicity, cell proliferation, cell morphology, and cell death (apoptosis/necrosis) were assessed in vitro. Results: MTA Flow™ White and ProRoot^®^ MTA leachates produced a strongly alkaline pH (~10–12) compared to the negative control, whereas MTA Flow™ leachate caused a smaller pH increase (~9.4). Freshly mixed cements showed moderate cytotoxicity (around 40–60% cell viability at 100% concentration), while hardened cement leachates did not significantly affect cell viability. At 100% concentration, MTA Flow™ and MTA Flow™ White leachates significantly inhibited hDPSC proliferation and caused cell death, but at lower concentrations (≤50%) they supported cell viability and proliferation comparable to ProRoot^®^ MTA. hDPSCs exposed to MTA Flow™ and MTA Flow™ White leachates appeared more elongated morphologically than those exposed to ProRoot^®^ MTA. Notably, cells treated with MTA Flow™ White leachates were significantly smaller than those treated with MTA Flow™. Conclusions: MTA Flow™ and MTA Flow™ White, used in 3:2 thick consistency, demonstrated biocompatibility comparable to ProRoot^®^ MTA in vitro. While 100% leachates showed moderate cytotoxicity, lower concentration dilutions (≤50%) supported hDPSC viability, proliferation, and morphology. These findings support their potential as safe alternatives for vital pulp therapy. Further in vivo studies and dynamic models are needed to confirm long-term biological performance.

## 1. Introduction

Vital pulp therapy (VPT), particularly direct pulp capping, is an essential endodontic procedure aimed at maintaining pulp vitality and promoting dentin bridge formation after pulp exposure [1]. The success of VPT depends on selecting a capping material that is biocompatible [2], bioactive [3], and promotes pulp healing [4]. Hydraulic calcium silicate-based cements (HCSC) have become the materials of choice for these procedures due to their superior sealing ability, antibacterial properties, and ability to stimulate mineralized tissue formation [5]. Both the European Society of Endodontology (ESE) and the American Association of Endodontists (AAE) recommend HCSCs [6,7], particularly mineral trioxide aggregate (MTA), as the gold standard for direct pulp capping due to their favorable biological response and clinical success rates [8]. Also, HCSCs are currently a choice of material for management of internal and external root resorption, closure of perforations [9], apexification [10], regenerative endodontic treatment [11,12], root-end filling [13], and root canal obturation [14].

These broad indications of HCSCs are due to their promising physicochemical and biological characteristics, such as the formation of calcium hydroxide on hydration, which leads to alkalinization of the environment with associated interactions [5], long-lasting ion release [15], or biological activity [16]. HCSCs can be classified according to their constitution or clinical use [17]. When used clinically, HCSCs interact with local tissue fluids, subsequently altering the local environment, increasing pH, and releasing calcium ions into the surrounding tissue, which is directly related to the effect on the surrounding tissues [18], for example, inducing cell differentiation [19]. After mixing HCSC or its introduction into contact with water, a hydration process occurs, resulting in the formation of tricalcium silicate and calcium hydroxide as a by-product. Further dissociation of calcium hydroxide creates a highly alkaline environment [20]. Furthermore, additives to the hydraulic calcium silicate-based cement, for example, silicon oxide (silica), can affect antimicrobial properties and enhance the biocompatibility of the material [19] by plateauing calcium ion release [21]. Finally, HCSCs were shown to liberate various bioactive materials from the tooth dentine matrix, including growth factors, such as TGF-β1 [22]. These bioactive molecules can modulate the cascade of various cellular events essential for pulp healing [23], such as cell attachment, differentiation, and cell migration [1].

As the behavior and properties, and especially the hydration process, of a hydraulic cement depend on its chemistry, the constitution of the material becomes critically important. Therefore, it is important to use reputable materials that have been adequately researched [24]. Currently, the most widely tested and gold standard are Portland cement-based mineral trioxide aggregate (MTA) dental materials [25]. Despite their shown biocompatibility [2] and bioactivity [3] benefits, HCSCs have some drawbacks in clinical practice, especially Type 1 to Type 3 Portland cement-based HCSCs [17]. These include the long setting time of Portland cement-based materials [26], discoloration potential when bismuth oxide is used as a radiopacifier, and tooth discoloration under certain conditions, such as blood contamination or exposure to specific irrigants, even when using tooth-colored HCSCs [14,27,28]. The iron present in hematin and hemoglobin can cause tooth color changes by infiltrating the dentine tubules [29], penetrating the pores and gaps of HCSC [30,31], or even being absorbed into freshly mixed HCSC [30]. Furthermore, sodium hypochlorite, when in contact with dental materials containing bismuth oxide as a radiopacifier, induces dark brown discoloration. This discoloration is attributed to oxidative degradation and phase transformation of bismuth oxide. Bismuth oxide can exist in multiple oxidation states (e.g., Bi_2_O_3_, Bi_2_O_4_, Bi_4_O_7_), and under oxidizing conditions such as exposure to NaOCl, these forms may transform and degrade, leading to the formation of dark-colored precipitates and bismuth carbonate. These chemical changes contribute to varying degrees of discoloration [32,33,34]. Even more, the initial long setting time may lead to decreased initial strength and increased early washout when placed in a wet operative field [35,36]. Finally, materials with a putty consistency can be challenging to handle and require direct visualization of the operative field [26]. To this end, a considerable amount of research has been undertaken to develop modified materials that have variable consistencies that are easy to use to suit the clinical need.

ProRoot^®^ MTA (Dentsply Tulsa Dental, Tulsa, OK, USA) is the first Portland cement-based dental material, which has been widely analyzed in preclinical studies and is known for its biocompatibility [37], antimicrobial activity [38], bioactive properties [39], and acceptable physicochemical characteristics [40]. Also, the material has been extensively tested in clinical studies with high success rates in vital pulp therapy after a 1-year follow-up period [41,42].

Recently, along with many other hydraulic calcium silicate types of cement, MTA Flow^TM^ and MTA Flow^TM^ White (Ultradent Products, Inc., South Jordan, UT, USA) were introduced into the market. These materials are Type 4 HCSC [17] and are mainly composed of tricalcium silicate, dicalcium silicate, and calcium sulfate. MTA Flow^TM^ contains bismuth oxide as a radiopacifier, while MTA Flow^TM^ White includes tantalum oxide to overcome tooth discoloration. The materials MTA Flow™ and MTA Flow™ White, according to the manufacturer, consist of ultrafine particles that may enhance the hydration reaction rate, and consequently, the increased exposed surface area would elevate the pH and increase the calcium ion release [43]. Although the MTA Flow^TM^ materials are relatively new in the market, several studies have analyzed the final irrigation protocol’s influence on the dislodgement resistance [44] and push-out bond strength [45], the MTA Flow^TM^ White’s thin consistency physicochemical, antimicrobial, and biological properties [46], and the inflammatory tissue response and mineralization ability of MTA Flow^TM^ [47]. MTA Flow™ is mixed with water-soluble silicone-based gel, allowing for a customizable gel-powder ratio to achieve various consistencies: 3:1 (thin), 3:2 (thick), and 1:1 (putty). This versatility facilitates their application in different clinical contexts. A thinner consistency can be delivered to hard-to-reach areas using a syringe when direct visualization is not feasible, while the putty consistency is suitable for procedures where direct observation is possible. While these modifications enhance practicality, their biological effects on human dental pulp stem cells (hDPSCs) have not been extensively studied, particularly at the 3:2 water-to-powder ratio consistency indicated for VPT. Innovative root-end filling materials based on calcium silicates and calcium phosphates have demonstrated promising physicochemical and biological performances. Abedi-Amin et al. compared experimental Portland-based and light-curing calcium silicate/phosphate cements, showing that their tested materials achieved high apical sealing ability, supported by mineral precipitation in simulated body fluid, and excellent cytocompatibility within 1–7 days [48]. Their findings support the relevance of material microstructure and ion release in driving both sealing and cellular responses, reinforcing the rationale behind comparative biocompatibility assessment of MTA Flow^TM^ on hDPSC.

This study aimed to evaluate the 3:2 liquid-to-powder ratio (thick) consistency of MTA Flow™ and MTA Flow™ White by examining their cytotoxicity, effects on proliferation, morphology changes, and induced cell death mechanisms in hDPSCs, in comparison to ProRoot^®^ MTA White.

## 2. Materials and Methods

### 2.1. Groups and Tested Materials in the Study

Groups in the study were divided as follows:

- Negative control group (NegativeCG)—growth medium. Serving as a reference control.
- Positive control group (PositiveCG)—leachates extracted from intermediate restorative material (IRM, Dentsply Tulsa Dental, Tulsa, OK, USA). A control group to ensure induction of apoptosis or necrosis.
- Control HCSC group (ProRootCG)—leachates extracted from gold-standard HCSC in research studies and dental clinical practice ProRoot^®^ MTA (Dentsply Tulsa Dental, Tulsa, OK, USA).
- Tested HCSC groups:
  o MTA Flow^TM^ group (MF)—leachates extracted from MTA Flow^TM^ 3:2 liquid-to-powder ratio consistency (Ultradent Products, Inc., South Jordan, UT, USA).
  o MTA Flow^TM^ White group (MFWhite)—leachates extracted from MTA Flow^TM^ White 3:2 liquid-to-powder ratio consistency (Ultradent Products, Inc., South Jordan, UT, USA).

A series of 100%, 50%, 25%, and 12.5% dilutions of PositiveCG, ProRootCG, and MF, MF White group leachates were used in this study. Table 1 features the hydraulic calcium silicate cement materials used in the study.

### 2.2. Cell Culture

The human dental pulp stem cells (hDPSCs) used in this study were obtained from a single, commercially available donor (Poietics^TM^ human dental pulp stem cells (hDPSCs), Lonza, Walkersville, MD, USA, Cat. No. PT-5025). Cells were pre-characterized by the supplier and validated for mesenchymal stem cell surface markers and differentiation potential. The cells were cultured according to the manufacturer’s protocol. Briefly, hDPSCs were maintained in growth medium (GM): alpha-medium essential (αMEM, Gibco, Thermo Fisher Scientific, Waltham, MA, USA, catalogue no. 12561056) supplemented with 10% fetal bovine serum (FBS; Gibco, Thermo Fisher Scientific, Waltham, MA, USA, catalogue no. A3160802) and 1% streptomycin/penicillin (Gibco, Thermo Fisher Scientific, Waltham, MA, USA, catalogue no. 10378016), cultured in a humidified incubator at 37 °C with 5% CO_2_. The hDPSC monolayer was detached by incubating cells for 3 min at 37 °C with EDTA-trypsin (0.25%) solution (Gibco, Thermo Fisher Scientific, Waltham, MA, USA, catalogue no. 25200056). Only passage number 3–5 cells were used in the experiments.

### 2.3. Material (Eluate/Leachate) Preparation

Hydraulic calcium silicate cements (HCSC) (MTA Flow^TM^ and MTA Flow^TM^ White) were prepared inside a laminar flow hood with sterile instruments following the respective manufacturer’s recommendations to the thick consistency: a ratio of 2 big ends of powder (0.26 g) and three drops of liquid. Briefly, 2.12 g in weight/1.3 mL in volume of the materials were prepared with the aid of a sterile 5 mL syringe and applied to the bottom of sterile 50 mm diameter glass plates by delivery tip (Micro Tip 20 Ga). The cement was compacted using a sterile cotton swab until homogenously distributed on the pre-marked plate surface area. All material preparations were performed by a single trained operator to reduce procedural variability and ensure consistency in the cement compaction process.

**Figure 1:**
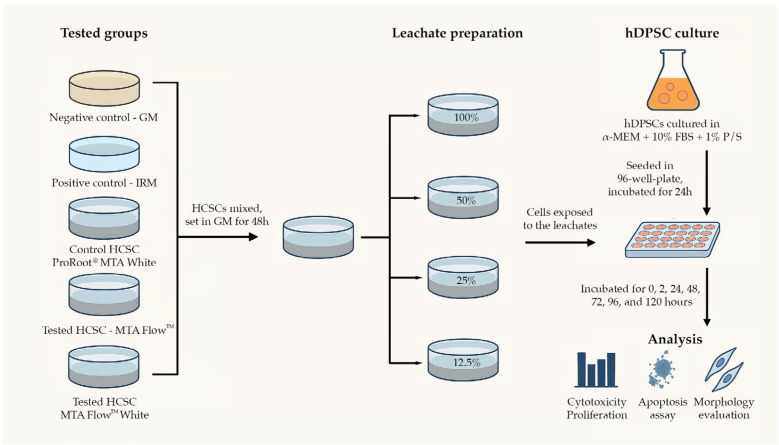
Schematic representation of the experimental workflow. Hydraulic calcium silicate-based cements (HCSCs) and control materials were prepared and incubated in growth medium (GM) for 48 h to produce leachates. These were diluted to 100%, 50%, 25%, and 12.5% concentrations. Human dental pulp stem cells (hDPSCs) were cultured in α-MEM with 10% FBS and 1% penicillin/streptomycin (P/S), seeded into 96-well plates, and exposed to leachates for time points ranging from 0 to 120 h. Cytotoxicity/proliferation (MTT assay), apoptosis/necrosis (annexin V), and morphology changes were assessed.

Immediately after preparing the cement, 13 mL of GM was added. The ratio between the surface of the cement exposed and the amount of liquid added was 1638 mm^2^ to 13 mL, or 126 mm^2^/mL, as stated in the ISO 10993-12:2021 standard [49]. The medium was left in contact with the disks for 48 h at 37 ◦C and 95% humidity in a CO_2_ free atmosphere with soda lime before being collected. The leachate was collected, centrifuged at 15,000 RPM (CL10 centrifuge, Thermo Scientific, Waltham, MA, USA), and filtered through the sterile filter with 0.22 µm size pores, and the 100, 50, 25, and 12.5% dilutions were made with GM. Eluates were immediately used for further experiments. A summary of the preparation and incubation parameters for cement leachate extraction is provided in Table 2. A schematic summary of the experimental methodology is presented in Figure 1.

### 2.4. pH Measurement

pH measurements of all 100% leachates were made in triplicate before centrifugation and filtration through a sterile filter with test papers (Johnson Test Papers Ltd., Tividale, UK). The pH of the leachates collected in GM, which consists of alpha-medium essential supplemented with 10% fetal bovine serum and 1% streptomycin/penicillin, was measured.

### 2.5. Cell Cytotoxicity and Proliferation

hDPSC (5000 cells/cm^2^) from third to fifth passages were seeded in a 96-well plate and incubated for 24 h. Afterwards, leachates were transferred onto hDPSC cultures and incubated for 0, 2, 24, 48, 72, 96, and 120 h. At each endpoint, GM was removed, and 100 μL of 3-(4,5-dimethylthiazol-2-yl)-2,5-diphenyltetrazolium bromide (MTT, Sigma-Aldrich Co., St. Louis, MO, USA) solution (0.5 mg/mL in GM) was added to each well, and the specimens were incubated for 3 h at 37 °C with 5% CO_2_. The MTT solution was discarded, and 100 μL of DMSO (≥99.5%, BioScience Grade, Thermo Fisher Scientific, Waltham, MA, USA) was added to each well. The resulting supernatant was then measured spectrophotometrically at 570 nm by a microplate reader (Varioskan^®^ Flash Multimode Reader, Thermo Scientific, Waltham, MA, USA). The results were expressed as a ratio in relation to the untreated negative control group cells. During the experiment, the leachates were renewed after three days by removing 50 μL of GM or leachate and adding 100 μL of new GM/leachate.

### 2.6. Annexin V Apoptosis Assay

To detect apoptotic hDPSCs after 24 h of incubation with 50% HCSCs leachates, annexin V conjugated with FITC and propidium iodide (PI) double staining was performed using the annexin V-FITC Apoptosis Detection Kit, according to the manufacturer’s instructions (Cat. No. 88-8005-72; eBioscience™, San Diego, CA, USA). The cells stained with annexin V-FITC and PI were analyzed with a flow cytometer (BD Bioscience FacsCanto II Flow Cytometer, Franklin Lakes, NJ, USA) using FCS Express version 7 (De Novo Software, Los Angeles, CA, USA) to read and analyze the effects of material extracts on the viable, necrotic, early, and late apoptotic cell ratios. The three cell viability experiments were repeated independently from each other, and the average of the obtained values was evaluated.

### 2.7. Cell Morphology Assessment

hDPSC (10 000 cells/cm^2^) of the third passage were seeded in 96-well plates and incubated for 24 h. After 24 h, leachates were transferred onto hDPSCs and incubated for 0, 2, 24, 48, 72, 96, and 120 h. At each endpoint, the effects of leachates from HCSCs on cell morphology changes were observed under an inverted phase contrast microscope (Olympus IX51, Olympus Corporation, Tokyo, Japan). Differences in cell morphology were evaluated by measuring cell width, length, and the ratio between length and width using the image processing program ImageJ (ImageJ 1.8.0_172, National Institutes of Health, Bethesda, MD, USA), as described previously by Alksne et al. [50].

### 2.8. Statistical Analysis

Data normality was evaluated using the Shapiro–Wilk test (normality assumption was met). The homogeneity of variances was assessed using Levene’s test. The outliers were identified by plotting boxplot graphs. Three-way and two-way ANOVA were performed to determine the interaction between HCSC type, leachate concentration, and/or exposure time. All simple pairwise comparisons were run with TukeyHSD for all HCSC leachate concentrations and all time points with Bonferroni adjustments applied. In graphs, the bar heights represent means, with error bars representing the standard deviations (SD). Statistically significant differences between the groups were marked as follows: *—*p* < 0.05, **—*p* < 0.01, ***—*p* < 0.001 and ****—*p* < 0.0001. Each cement leachate was analyzed in triplicate per test. IBM SPSS Statistics, version 29 (IBM Corp., Armonk, NY, USA) and GraphPad Prism, version 9 (GraphPad Software, San Diego, CA, USA) were used for statistical analysis.

## 3. Results

### 3.1. Characteristics of the Leachates

**Figure 2:**
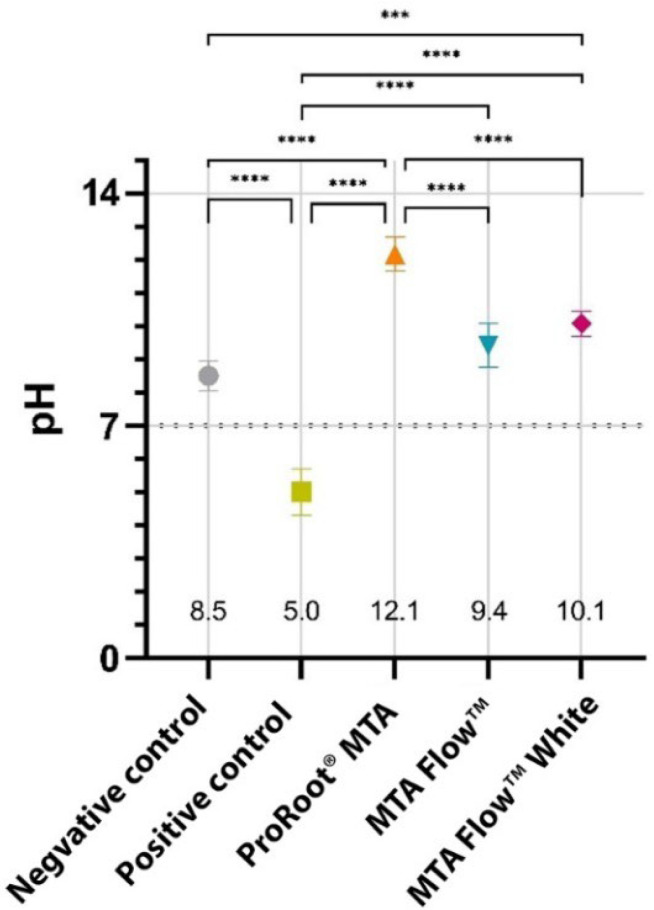
Freshly extracted HCSCs 100% leachates mean pH values. *—marks statistically significant differences between the groups (***—*p* < 0.001 and ****—*p* < 0.0001).

All tested HCSC groups had an alkaline effect, while PositiveCG had an acidic effect on the pH of the growth medium. Freshly extracted HCSC 100% leachate pH values of ProRootCG (12.1), MFWhite (10.1), and ProRootCG (5.0) were statistically significantly different from NegativeCG (8.5; *p* < 0.05). However, MF resulted in a slightly smaller pH alteration (9.4) than MFWhite, which was not statistically significantly different from NegativeCG (*p* > 0.05). The pH of the MF and MFWhite leachates were comparable between the groups (*p* > 0.05); however, they were statistically significantly different from ProRootCG, which resulted in the most alkaline pH (12.1; *p* < 0.05). Detailed pH values of freshly extracted HCSCs 100% leachates and their comparison can be seen in Figure 2.

### 3.2. MTA Flow^TM^ and MTA Flow^TM^ White Cytotoxicity Assay

**Figure 3:**
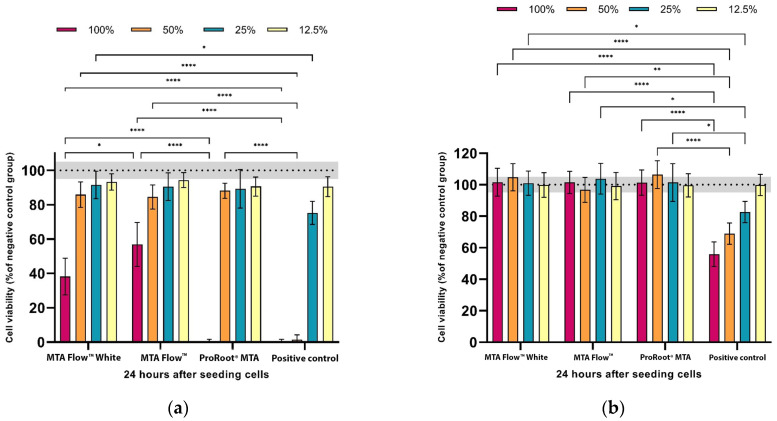
MTT assay: effect of the leachates with different dilutions after 24 h of exposure on hDPCs: (**a**) the leachates derived from the freshly mixed MTA Flow^TM^ and MTA Flow^TM^ White; (**b**) the leachates derived from the hardened MTA Flow^TM^ and MTA Flow^TM^ White. The results are presented as a percentage of the negative control group (100% reference line with ±SD grey box). *—marks statistically significant differences between the groups (*—*p* < 0.05, **—*p* < 0.01 and ****—*p* < 0.0001).

Cell viability was significantly affected by the presence of leachates from freshly mixed cement and to different degrees depending on the cement-leachate solution. Hardened HCSC leachates at all concentrations did not influence cell viability (*p* > 0.05). The cell-viability data of hDPSCs exposed to freshly mixed and hardened HCSCs are summarized in Figure 3.

At 100% concentration, freshly mixed PositiveCG and ProRootCG resulted in complete cell death. While the same concentration of MF and MFWhite groups reduced cell viability to 57 ± 13% and 38 ± 10% compared to NegativeCG, respectively. Moreover, no statistically significant differences were found between MF and MFWhite leachates at all concentrations after 24 h (*p* < 0.05). Both MF and MFWhite groups were moderately toxic to hDPSCs; however, both cements 100% leachates were significantly less cytotoxic to hDPSCs compared to ProRootCG 100% leachate (*p* < 0.05).

At lower concentrations, including 50%, 25%, and 12.5%, the viability of tested HCSC groups remained above 80% with no statistically significant differences between the HCSC groups. On the other hand, no viable cells were observed in a PositiveCG at 100% and 50% dilution, and statistically significant reduced cell viability to 75 ± 7% at 25% dilution compared to NegativeCG (*p* < 0.05).

When testing leachates collected from hardened HCSCs, no differences were found between the tested cement groups compared to NegativeCG (*p* > 0.05). Only PositiveCG significantly reduced cell viability to 55 ± 8%, 69 ± 7%, and 83 ± 7% compared to NegativeCG, depending on the concentration (*p* < 0.05).

### 3.3. hDPSCs Proliferation Assay

The proliferation of hDPSCs was significantly affected by the presence of the freshly mixed cement leachate. All main effects and interaction terms demonstrated statistically significant contributions with large effect sizes, as indicated by partial eta squared (η^2^) values > 0.97. Notably, the Time × Cement × Concentration interaction had a partial η^2^ of 0.987 (95% CI [0.296–0.421]), highlighting that the cellular response was strongly dependent on the combined influence of material type, its concentration, and exposure duration. NegativeCG cell proliferation steadily increased from 1 ± 0.1 at 2 h to a 10.6 ± 0.1-fold increase in cell number at 120 h. The 100% and 50% leachates from PositiveCG induced cell death, with no observed hDPSC proliferation. Lower concentrations had a proliferation-inhibiting effect: cells treated with 25% and 12.5% leachate solutions proliferated 3.8 ± 0.1 and 7.4 ± 0.1 times, respectively, over 120 h (*p* < 0.05). Statistical significance was accepted at the *p* < 0.017 level for simple two-way interactions and simple-simple main effects. Detailed results of three-way ANOVA showing effect sizes for the influence of time, cement type, and leachate concentration on cell proliferation can be found in Table 3.

Overall, all tested HCSC leachates reduced cell proliferation at all times when exposed to 100% and 50% concentrations compared to NegativeCG (*p* < 0.017). However, the strength of the inhibitory effect of the leachates depended on their concentration. At 100% leachate concentration, the ProRootCG had a similar proliferation pattern to that of PositiveCG at all time points by inducing cell death (0.1 ± 0.1). Both MF and MFWhite leachates caused hDPSCs death and/or inhibited proliferation, yet cell viability remained higher, ranging from 0.3 ± 0.1 to 1 ± 0.2 times in MF and from 0.6 ± 0.1 to 0.7 ± 0.1 in MFWhite groups, compared to the PositiveCG and ProRootCG (*p* < 0.017). Proliferation remained significantly higher from 24 h onward in the 50% and 25% HCSCs groups compared to the PositiveCG (*p* < 0.017).

There was a statistically significant simple-simple main effect of HCSC type for hDPSC proliferation at all concentrations and time points. At 50%, all tested HCSCs resulted in steady cell proliferation from 2 to 120 h, depending on the group: ProRootCG increased from 0.8 ± 0.1 at 2 h to 4.6 ± 0.2 times at 120 h, MF cell proliferation increased from 0.5 ± 0.1 to 4.4 ± 0.1 times, and MFWhite increased from 0.9 ± 0.1 to 4.6 ± 0.2 times the change in cell number, respectively. At 25% concentration, the proliferation rates of all tested HCSC groups were higher from 2 to 120 h, ranging from 0.8 ± 0.1 to 7.5 ± 0.1 in ProRootCG, from 0.6 ± 0.1 to 7.5 ± 0.1 in MF, and from 0.9 ± 0.1 to 8 ± 0.1 times in the MFWhite group. Finally, at 12.5%, the proliferation rate increased more, resulting in a final hDPSC proliferation rate of around 9.5 ± 0.1 in all HCSC groups at 120 h (*p* > 0.017).

At 2 h, MF’s 100% leachate significantly reduced hDPSCs proliferation to 0.3 ± 0.1 compared to MFWhite at 0.6 ± 0.1 cell changes in number (*p* < 0.017). From 24 h onward, MF group proliferation was about 0.07 to 0.37 times higher than MFWhite or similar (*p* < 0.017). This pattern persisted at 50% and 12.5% concentrations at 48 h, with MFWhite showing 0.3 and 0.2 times lower proliferation, respectively (*p* < 0.017). However, at 48 h, the 25% difference between the groups was not significant (*p* > 0.017). At 120 h, 25% MF and ProRootCG had 7.5 ± 0.1 times proliferation, while 25% MFWhite resulted in 8 ± 0.1 times proliferation (*p* < 0.017).

**Figure 4:**
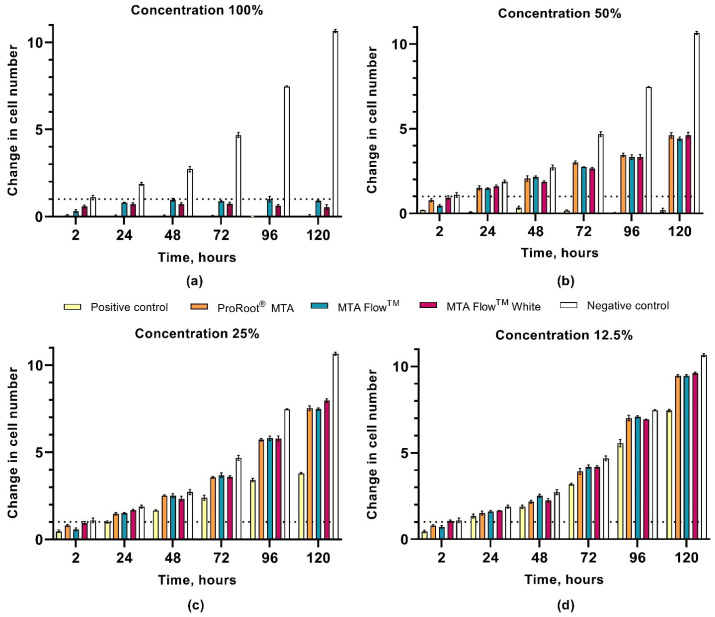
The effect of the leachates derived from the freshly mixed HCSCs tested at different time points on the proliferation of hDPCs with varying factors of dilution: (**a**) 100%; (**b**) 50%; (**c**) 25%; (**d**) 12.5%. Results are presented as change in cell number according to the 2 h NegativeCG as a reference point. A pairwise comparison analysis of each group can be found in the Supplementary Materials Tables S1–S4.

All simple pairwise comparisons were run for all HCSC leachate concentrations and all time points with a Bonferroni adjustment applied. The proliferation rates of hDPCs exposed to HCSCs leachates for 2, 24, 48, 72, 96, and 120 h are summarized in Figure 4a–d, according to the 2 h NegativeCG (reference point). hDPSCs proliferation means [CI] and pairwise comparisons according to the group, concentration, and time can be found in Supplementary Materials Tables S1–S4.

### 3.4. Human Dental Pulp Stem Cells Morphology Assessment

**Figure 5:**
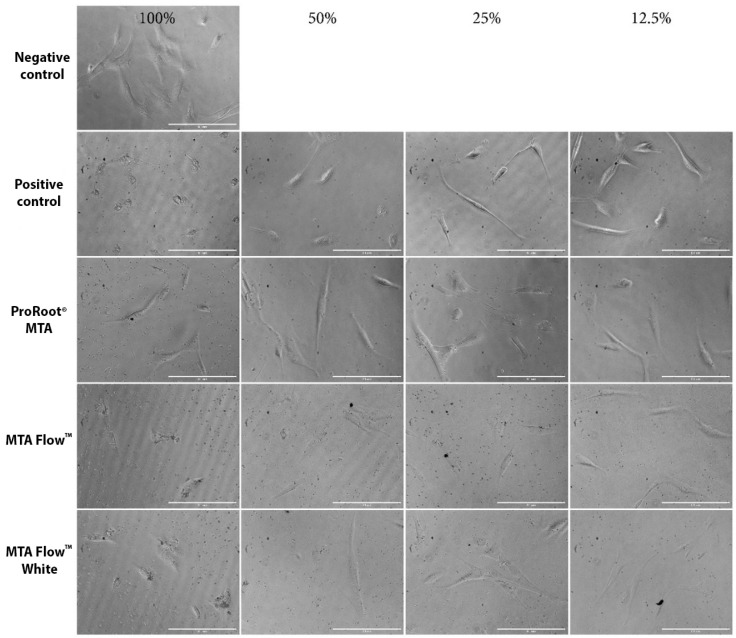
Representative hDPSC morphology assessment images after incubation with freshly mixed HCSC leachates. Alongside, hDPSC morphology after incubation with freshly mixed control HCSC (ProRoot^®^ MTA), positive and negative control groups.

**Figure 6:**
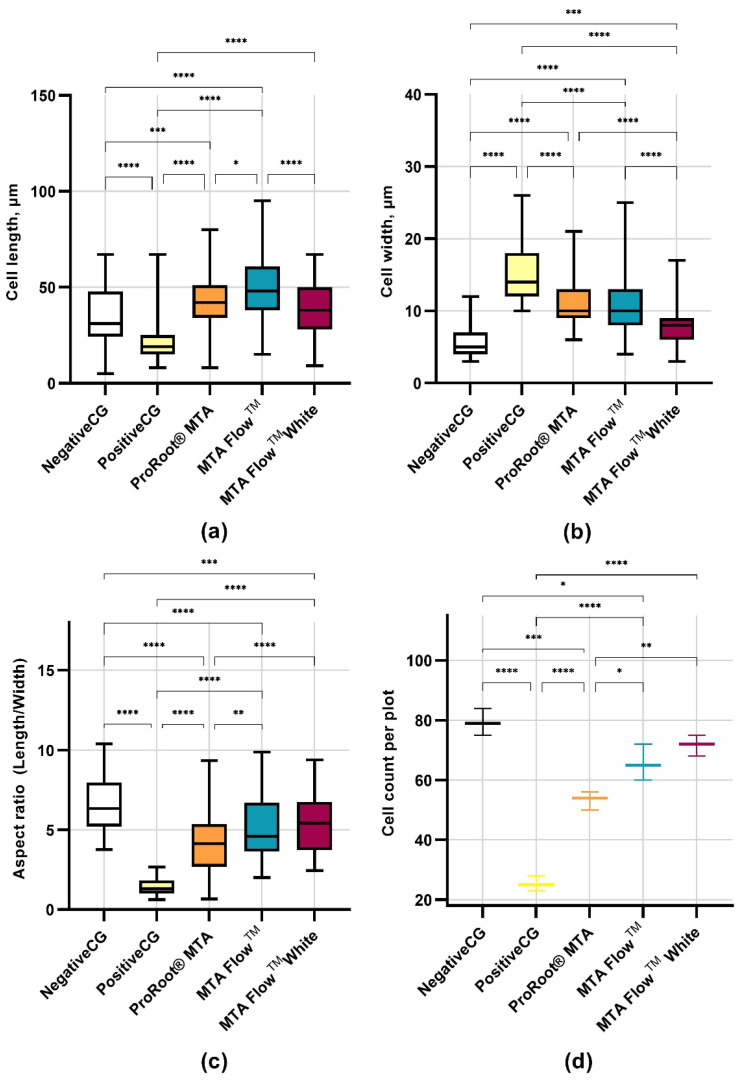
Assessment of hDPSCs morphology and cell mean count per standardized frame after incubation for 24 h with freshly mixed HCSCs 50% leachates and control groups: (**a**) Assessment of hDPSCs length, in µm; (**b**) Assessment of hDPSCs width, in µm; (**c**) Assessment of hDPSCs aspect ratio (cell length divided by cell width); (**d**) Assessment of hDPSCs count per frame. *—marks statistically significant differences between the groups (*—*p* < 0.05, **—*p* < 0.01, ***—*p* < 0.001 and ****—*p* < 0.0001).

hDPSCs morphology was evaluated using an inverted phase contrast microscope after cell treatment with different concentrations of HCSCs leachates, while quantitative hDPSCs morphology analysis was performed after incubation with 50% HCSCs MF, MFWhite, ProRootCG, PositiveCG, and NegativeCG. In the NegativeCG, hDPSCs were spindle-shaped and spread over the entire plate well surface area (Figure 5 and Figure 6a–d). Also, it contained pale, round, or oval central nuclei with multiple nucleoli. In contrast to the NegativeCG, the 100% tested HCSC groups at all monitored time points determined more round cell morphology and decreased cell number. Moreover, after treatment with 50% HCSC leachates, the cell length and width were larger than in NegativeCG. However, after incubation of hDPSCs with 25% HCSC leachates, cell morphology remained almost the same as in NegativeCG, although cells were less frequently spread on the surface.

The PositiveCG group had the smallest cell length (22.3 ± 11.2 µm) and highest width (15.1 ± 3.8 µm), resulting in the most rounded cell shape (aspect ratio 1.4 ± 0.5) and fewest in number (25.3 ± 2.5 cells/frame), while the mean cell length of NegativeCG was 34.6 ± 13.7 µm, width −5.6 ± 2.3 µm, and resulted in 6.6 ± 1.6 cell aspect ratio. hDPSCs exposed to 50% MF leachate were longer (48.9 ± 16.4 µm) than those exposed to 50% MFWhite (39.6 ± 14.1 µm) and ProRootCG (43.1 ± 13.9 µm); however, no significant length differences were found between ProRootCG and MFWhite. The MFWhite group had the narrowest cells of all HCSC groups (7.7 ± 2.4 µm), while similar widths were found in MF (11.0 ± 4.5 µm) and ProRootCG (10.8 ± 2.8 µm). ProRootCG revealed a 4.3 ± 1.8 cell aspect ratio and 53.3 ± 3.1 cell count number per frame, while MF and MFWhite had 5.1 ± 2.0 and 5.6 ± 1.9 cell aspect ratios and 65.7 ± 6.0 and 71.7 ± 3.5 cell count numbers, respectively. There was no difference in cell aspect ratios between the MF and MFWhite groups. Overall, hDPSCs treated with all HCSCs groups leachates were longer and wider (lower aspect ratio) and had lower cell numbers than in the NegativeCG.

### 3.5. Annexin V Apoptosis Assay

**Figure 7:**
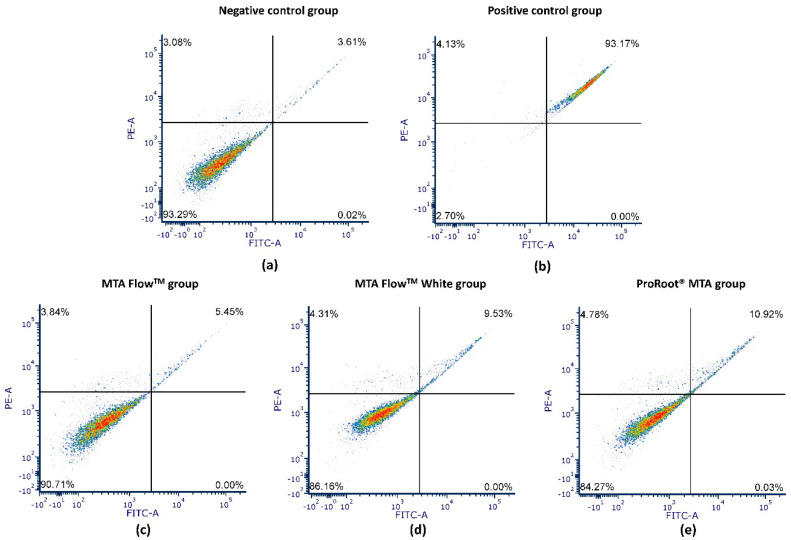
Representative dot plot images of flow cytometry annexin V-FITC/PI apoptosis assay of hDPSCs treated with 50% leachates for 24 h and control groups. Subfigures show: (**a**) Negative control group (NegativeCG); (**b**) Positive control group (PositiveCG); (**c**) MTA Flow™; (**d**) MTA Flow™ White; and (**e**) ProRoot^®^ MTA. In each plot, the quadrants represent cell populations as follows: lower left – viable cells (Annexin V−/PI−), lower right – early apoptotic cells (Annexin V+/PI−), upper right – late apoptotic cells (Annexin V+/PI+), and upper left – necrotic cells (Annexin V−/PI+). Color intensity represents cell density, with blue indicating the lowest, green - intermediate, and red - the highest cell density.

Flow cytometry analysis was performed on hDPSCs treated with 50% HCSCs MF, MFWhite, ProRootCG, PositiveCG, and NegativeCG (Figure 7). The flow cytometry graphs show live cells (lower left), early apoptotic cells (lower right), late apoptotic cells (upper right), and necrotic cells (upper left).

All tested HCSCs, including MF, MFWhite, and ProRootCG, had 50% leachates with over 84% viable cells after 24 h. In contrast, PositiveCG reduced viability to 4.5%, while NegativeCG maintained around 86.5 ± 6.1% viable cells. There were no significant differences between NegativeCG and all tested HCSCs with 50% leachates in terms of viable and late apoptotic cell ratios, nor were there significant differences in necrotic cell ratios. Detailed analysis of annexin V-FITC apoptosis/necrosis flow cytometry results is presented in Table 4 and Figure 7.

## 4. Discussion

The investigation of biological characteristics in vitro using cell cultures is a crucial step in analyzing HCSC materials. These controlled laboratory tests provide valuable insights into potential cytotoxic effects on the tested cell cultures. Thus, the findings of this study provide important insights into the biological properties of MTA Flow™ and MTA Flow™ White when used at a 3:2 liquid-to-powder ratio (thick consistency). The study compared their effects on hDPSCs in terms of cytotoxicity, proliferation, morphology changes, and cell death mechanisms relative to ProRoot^®^ MTA, a well-established hydraulic calcium silicate cement (HCSC).

Most in vitro studies focus on assessing the impact of eluates from hardened HCSC materials on cell cultures [1,51,52,53]. Analyzing the eluates from hardened HCSC is crucial for understanding the long-term effects on cells and periapical tissues. However, it is essential to note that only freshly mixed cements or sealers, still in the setting phase, are introduced into dental operative sites [54]. Freshly mixed HCSC materials interact with environmental fluids and surrounding tissues, including dentine, releasing calcium ions and leachable components [5,55]. This initial interaction raises pH levels and can have a pronounced impact on surrounding cells [5,20,21,56,57]. Once the material hardens, the interaction with dental pulp tissues continues, even though the pH is buffered and byproducts form [58].

Our study confirmed these observations by comparing leachates from freshly mixed and hardened HCSC materials. The cytotoxicity of hardened cement leachates was negligible across all tested HCSC materials, while freshly mixed cement eluates, even at 25% dilutions, resulted in significantly lower cell viability. Therefore, once the HCSC materials are set, they are biocompatible and unlikely to cause significant adverse effects on surrounding pulp tissue. These findings align with previous studies demonstrating that cytotoxicity of calcium silicate-based cements is predominantly observed in freshly mixed materials and is reduced after setting [58,59]. Consequently, recent studies have modified their methodologies to analyze the biocompatibility of freshly mixed HCSC leachates [46,54]. Thus, this study employed both methodologies to compare the effects of freshly mixed and hardened HCSC eluates on hDPSC cytotoxicity after 24 h of incubation. Upon detecting a significant impact of freshly mixed HCSC eluates on hDPSCs, we proceeded with further analysis using this approach.

When HCSC materials are placed on the dental pulp, calcium hydroxide leaches into the surrounding tissues upon hydration, leading to the alkalinization of the environment. This process has implications for clinical interactions and the formation of surrounding leachate [5]. The pH analysis revealed that all tested HCSC materials produced an alkaline environment, with ProRoot^®^ MTA exhibiting the highest pH of 12, consistent with previous research indicating its strong alkalizing properties [60]. Both MTA Flow™ and MTA Flow™ White exhibited a lower pH, yet were still alkaline. pH analysis results are similar to those of the study by B. Guimares et al., who found MTA Flow^TM^ pH after 24 h to be around 10 [61]. The slightly lower pH of MTA Flow™ materials could be attributed to their composition, particularly the presence of calcium sulfate and tantalum oxide in MTA Flow™ White instead of bismuth oxide. Given that an alkaline pH plays a critical role in antibacterial effects and tissue healing, the results suggest that both MTA Flow ™ materials retain an environment conducive to these processes while potentially reducing the severe alkalinity-related cytotoxicity observed with ProRoot^®^ MTA [62].

Direct contact evaluations with HCSC materials require material sterilization, which may affect their properties [58]. While concerns about specimen contamination during leachate preparation have been raised [54], sterile instruments and filters were used in a laminar flow cabinet to ensure sterility. Varying leachate concentrations allowed for the analysis of dose-related relationships and the determination of the ideal concentration for hDPSCs’ sensitivity [58,63].

According to the ISO 10993-5:2009 standard, cytotoxicity is determined when cell viability falls below 70% [64]. The results of this study confirm a clear dose-dependent cytotoxicity profile for all tested HCSCs, with lower leachate concentrations (≤50%) maintaining viability above the ISO 10993-5 [64] cytotoxicity threshold of 70%, while 100% leachates showed moderate to severe cytotoxicity. After 24 h of incubation with 100% HCSC leachates, cell viability was approximately 40% in the MTA Flow™ White and about 50% in the MTA Flow™ group, in contrast to the negative control group. The pronounced cytotoxicity observed in 100% eluates may also be partially attributed to their elevated alkalinity. The pH levels of undiluted leachates, particularly from ProRoot^®^ MTA, exceeded 12, which surpasses physiological limits tolerated by hDPSCs. At such high pH values, cellular membrane integrity and metabolic activity may be compromised, potentially explaining the significantly reduced viability compared to diluted groups [65]. However, their effects at lower concentration dilutions (25–50%) showed cell viability above the threshold, supporting conditional biocompatibility depending on concentration and setting phase. The initial inhibition of hDPSC proliferation by HCSC has been reported in both in vitro [54,66] and in vivo studies [67]. Despite the lower initial cell viability compared to the control group, the use of HCSC cement/sealers in vivo has indicated high clinical success rates for primary root canal treatment, reaching around 89% after 36 months [68,69].

Interestingly, MTA Flow™ White showed higher cytotoxicity at 100% concentration compared to MTA Flow™. This might be related to the inclusion of tantalum oxide—while generally considered cytocompatible and bioactive, as evidenced by increased ALP activity and mineralization in studies of tantalum-containing cements [70,71], its unique microstructure or ion release profile may impact early cell viability in high-concentration eluates. Further studies are needed to investigate whether tantalum-containing compounds influence oxidative stress or cellular metabolism in dental pulp cells.

Moreover, similar or higher cell viability, maintaining above 80% compared to the control group, was observed after 24 h of incubation with 50%, 25%, and 12.5% concentrations of MTA Flow ™ and MTA Flow ™ White eluates. In contrast, statistically significant differences were found between the groups affected by MTA Flow ™ leachates and ProRoot^®^ MTA leachate, with cell viability around 0%, indicating severe cytotoxicity on hDPSCs and no significant difference from the positive control group. These results are consistent with a previous study by Chawan Maspon et al., which also reported similar cytotoxicity of 100% ProRoot^®^ MTA leachate after 24 h [1]. The higher cytotoxicity observed for ProRoot^®^ MTA at 100% may be attributed to its slower setting time, higher pH, and the presence of bismuth oxide, which has been associated with less favorable biological responses compared to tantalum-based radiopacifiers used in MTA Flow ™ White [72]. Although ProRoot^®^ MTA showed higher cytotoxicity at 100%, its lower concentration leachates maintained hDPSC viability above 80%, supporting the interpretation that similarly diluted MTA Flow ™ and MTA Flow ™ White leachates are also biocompatible. [1,73,74,75].

The annexin V apoptosis assay confirmed the biocompatibility of MTA Flow ™ and MTA Flow ™ White, with over 84% of hDPSCs remaining viable after 24 h of exposure to 50% leachates. This was comparable to ProRoot^®^ MTA and significantly higher than the positive control, which induced substantial apoptosis and necrosis. These findings suggest that at lower concentrations (e.g., 50% leachates), both MTA Flow ™ materials support hDPSC viability without inducing significant apoptosis or necrosis, reinforcing their potential safety in clinically relevant applications. As HCSC is placed directly on a pulp, they initially create an alkaline environment that is bactericidal and stimulatory for healing [45]. It is also important to note that 100% leachate concentrations may not accurately reflect clinical exposure levels, especially after the material has begun to set and diffuse into surrounding tissues. As MTA Flow™ resulted in slightly lower initial pH and cytotoxicity than ProRoot^®^ MTA, it may cause less initial irritation to pulp tissue while still promoting healing. A moderate initial cell impact can be expected, leading to a transient inflammation seen in the in vivo scenario [4,76] that precedes healing and dentine bridge formation. Similarly, Abedi-Amin et al. highlighted that calcium silicate/phosphate cements facilitated mineral precipitation in mineralizing solutions and demonstrated favorable cytocompatibility, linking their bioactivity to physicochemical interactions with surrounding tissues [48]. This aligns with our observations where MTA Flow ™ induced sustained cell proliferation and may promote mineralizing effects in vitro.

The proliferation of hDPSCs was notably impacted by the freshly mixed HCSC leachates across all groups. However, the extent of the inhibitory effect varied depending on the concentration of the leachates. Several prior studies have investigated the biocompatibility of MTA Flow™ and MTA Flow™ White using diverse methodologies [46,47,77,78]. Bueno et al. [47] and Mondeli et al. [77] studied HCSC in rat subcutaneous tissue over various time points and reported a moderate inflammatory reaction to MTA Flow™ 3:2 liquid-to-powder ratio consistency at 7 days, diminishing over time. In another study, Pelepenko et al. assessed the cytotoxicity of MTA Flow™ thin consistency on periodontal ligament fibroblasts after 24 h [78], utilizing a clinically relevant 3D cell culture model associated with an in-situ root-end filling experimental setup [79]. Interestingly, the periapical model with periodontal ligament fibroblasts showed no cytotoxicity compared to the negative control group. Another study by Pelepenko et al. examined freshly mixed MTA Flow™ White thin consistency, yielding different results compared to our study due to distinct methodologies and cell cultures [46]. Our study demonstrated more severe and rapid cytotoxicity of 100% leachates. This disparity could be attributed to the pre-preparation of 100% leachates and the complete change of growth medium to 100% leachate after 24 h in our study, contrasting with the gradual medium pH changes observed in Pelepenko et al.’s 3D model. Additionally, hDPSCs, characterized as relatively slow-proliferating cells, exhibit different proliferation patterns compared to periodontal ligament stem cells [80]. Hence, variations in cell cultures and methodology for assessing the cytotoxicity of freshly mixed MTA Flow™ White could influence the outcomes. By indirectly comparing hDPSC proliferation results with viable, apoptotic, and necrotic cell analysis, the cytotoxicity of 100% leachates could be evaluated as mild to severe, respectively.

Analyzing hDPSC proliferation over five days revealed a time-dependent phenomenon: cell proliferation increased in all tested HCSC materials’ 50%, 25%, and 12.5% concentration leachate groups at later time points. The proliferation of hDPSCs affected by tested MTA Flow™ and MTA Flow™ White was similar to the ProRoot^®^ MTA cell group in 50%, 25%, and 12.5% concentrations at all time points. However, no increase in cell proliferation was observed in 100% leachate groups throughout the observation period. Only MTA Flow™ and MTA Flow™ White maintained cell viability around 5 to 10% compared to the negative control group. These findings align with previous studies where freshly mixed 100% leachate resulted in the lowest cell viability [1]. Therefore, reducing the eluate concentration and prolonged exposure to lower or equal to 50% concentration leachates led to higher cell viability.

The cell morphology in the negative control and tested groups affected by lower leachate concentrations resembled the characteristic elongated fibroblastic morphology of mesenchymal stem cells described previously [81,82]. Compared to the negative control group, hDPSCs affected by 100% leachate exhibited a more rounded cell morphology and decreased cell number. This altered morphology in cells exposed to 100% leachates may be influenced by extreme alkalinity, reinforcing the importance of dilution in maintaining a physiologically tolerable environment. Additionally, a concentration-dependent phenomenon was observed, where reducing the eluate concentration preserved morphology more similar to the negative control group. An interesting exception was observed at 50% concentration, where the morphology of hDPSCs affected by tested MTA Flow™ and MTA Flow™ White was longer and wider than that of the negative control group, though the differences were not statistically significant compared to ProRoot^®^ MTA. In the literature, hDPSCs cultured on the surface of MTA were shown to exhibit less fibrous and more differentiated morphology than the negative control group [83]. This could be attributed to the bioactive potential of HCSC on hDPSCs, as mentioned previously [23,84]. Interestingly, the width and length of hDPSCs affected by 50% MTA Flow ™ leachate were significantly higher than those affected by 50% MTA Flow ™ White, while the aspect ratio remained similar. Furthermore, cells affected by 50% MTA Flow ™ White leachate exhibited morphology more similar to the negative control group compared to the 50% MTA Flow ™ group. In contrast, previous studies have reported varying degrees of hDPSC shrinkage after exposure to different HCSC leachates [82] and superior spreading of the cells [85]. However, direct comparison is challenging as no previous studies have evaluated hDPSCs morphology after exposure to MTA Flow ™ or MTA Flow ™ White leachates. Importantly, despite minor morphological changes, cell count and viability remained high, suggesting that these materials do not induce severe cellular stress or damage [83]. The observed elongation and reduced cell width in response to MTA Flow ™ and MTA Flow ™ White may reflect alterations in cytoskeletal organization, potentially influencing cell migration or differentiation trajectories. These morphological adaptations suggest a cellular stress response or remodeling mechanism that warrants further investigation through molecular and migration assays.

However, the in vitro nature of this study introduces certain limitations; therefore, it’s crucial to critically evaluate these results. Firstly, changes in the water-to-powder ratio can lead to the deterioration of the physical properties of HCSC materials [86]. Therefore, current results can only be applied to the biological properties of MTA Flow ™ and MTA Flow™ White when used at a 3:2 liquid-to-powder ratio (thick consistency). Additionally, the leachate extraction period of 48 h, while ISO 10993-12 [49] compliant, may not fully reflect long-term ion release kinetics. Future studies should consider time-course analyses of ion composition and evaluate materials under dynamic exposure systems that better mimic clinical conditions.

Secondly, the use of static leachate conditions does not replicate the dynamic fluid environment found in vivo, where pulpal fluid flow and continuous exchange of metabolites may influence cellular responses. Particularly, isolated cell cultures lack the complexity of in vivo dental pulp tissues [87], which comprise a variety of cell types and are influenced by factors such as blood flow, buffering systems, and the immune response [88,89]. Moreover, buffering systems in the growth medium might influence leachates’ pH values and resulting hDPSC viability [90]. α-MEM typically utilizes a sodium bicarbonate buffering system, which maintains physiological pH levels (approximately 7.2 to 7.6) when incubated in a CO_2_-enriched environment (5–10% CO_2_) [91]. On the other hand, while α-MEM’s buffering system is designed for typical physiological conditions, its capacity to counteract alkaline challenges may be limited [92]. Finally, inflammation in the dental pulp can alter the clinical context by progressing apically over time [88].

Another limitation is the absence of surface characterization of the cements before and after leachate preparation. Analytical techniques such as scanning electron microscopy (SEM) or energy dispersive X-ray spectroscopy (EDS) could provide insights into material microstructure and ion release profiles. Also, while 2D morphological metrics such as cell length, width, and aspect ratio offer valuable information, they are limited in describing the complex cytoskeletal and spatial changes occurring at the cellular level. Future studies should incorporate fluorescent cytoskeletal staining or 3D imaging via confocal microscopy to gain a more detailed understanding of cell-matrix interactions. Moreover, the biological evaluation could be enhanced by, including molecular markers of osteogenic differentiation (e.g., ALP, RUNX2, DSPP) and inflammation (e.g., IL-6, TNF-α). These would provide mechanistic insights into how material-induced changes influence pulp healing processes.

Nevertheless, exposing cell cultures to HCSC leachates offers valuable insights into how these materials may interact with local tissues in vivo. Analyzing subsequent cellular responses can shed light on biocompatibility, cytotoxicity, proliferation, and differentiation. Consequently, our study indicates comparable biocompatibility between the new generation HCSC materials MTA Flow^TM^ and MTA Flow^TM^ White 3:2 liquid-to-powder ratio consistency and the original ProRoot^®^ MTA White when tested with hDPSC cultures with slightly reduced cytotoxic effects in freshly mixed states. The versatility of these materials, particularly their ability to be mixed at different consistencies, makes them promising candidates for various clinical applications, including vital pulp therapy and root-end filling. Further in vivo studies are necessary to confirm their long-term biocompatibility and clinical performance, but these in vitro findings provide strong preliminary evidence supporting their use as bioactive endodontic materials.

## 5. Conclusions

MTA Flow ™ and MTA Flow ™ White, when used at a 3:2 powder-to-liquid ratio (thick consistency), demonstrated comparable biocompatibility to ProRoot^®^ MTA White. All tested HCSC materials showed dose-dependent cytotoxicity: freshly mixed 100% leachates significantly reduced hDPSC viability, while lower concentration dilutions (≤50%) maintained cell viability above the ISO 10993-5 cytotoxicity threshold.

MTA Flow™ White exhibited slightly higher cytotoxicity than MTA Flow ™ at 100% leachate concentration, although both materials promoted similar proliferation and morphology at lower concentrations.

Despite initial cell stress at high leachate concentrations, all materials supported hDPSC proliferation over five days at lower concentrations and induced minimal apoptotic or necrotic cell death. These findings suggest that MTA Flow ™ and MTA Flow ™ White are biocompatible alternatives to ProRoot^®^ MTA for vital pulp therapy, particularly in applications requiring controlled consistency and aesthetic considerations.

Future research should include dynamic leachate models, long-term evaluations, and in vivo studies to confirm clinical biocompatibility and performance. Testing both freshly mixed and set material eluates remains essential for a comprehensive biological risk assessment of HCSC materials.

## Figures and Tables

**Table 1:** Materials and their composition were used in the study.

Material Type	Material	Powder Composition	Liquid	Manufacturer
Portland cement	ProRoot^®^ MTA	Portland cement, calcium sulfate dihydrate, tetracalcium aluminoferrite, gypsum, calcium oxide, bismuth oxide	Distilled water	Dentsply Tulsa Dental, Tulsa, OK, USA
Type IV HCSC	MTA Flow ^TM^	di- and tricalcium silicate, calcium sulfate, silica, bismuth trioxide	Water, Water-soluble silicone-based gel	Ultradent Products, Inc., South Jordan, UT, USA
	MTA Flow^TM^ White	di- and tricalcium silicate, calcium sulfate, silica, tantalum oxide		

**Table 2:** Overview of cement eluate preparation parameters.

Parameter	Description		
Cement Type	ProRoot^®^ MTA	MTA Flow™	MTA Flow™ White
Preparation consistency *	0.5 g powder + 0.167 mL liquid (putty consistency, 3:1)	0.26 g powder + 3 drops (0.17 mL) gel (thick consistency, 3:2)	
Total volume of material	1.3 mL		
Application surface	50 mm diameter sterile glass plates		
Material compaction	Homogeneously distributed using a sterile cotton swab		
Medium volume added	13 mL of growth medium		
Surface area-to-volume ratio	126 mm^2^/mL (compliant with ISO 10993-12:2021)		
Extraction conditions	48 h at 37 °C, 95% humidity, CO_2_-free incubator with soda lime		
Post-processing	Centrifuged (15,000 RPM), filtered (0.22 µm), diluted to 100%, 50%, 25%, 12.5%		
Extraction medium	α-MEM + 10% FBS + 1% penicillin/streptomycin		

* Materials were prepared according to the manufacturer’s instructions.

**Table 3:** Results of three-way ANOVA showing effect sizes for the influence of time, cement type, and leachate concentration on cell proliferation.

Effect	F (DFn, DFd)	Partial η^2^	95% CI
Cement type	F(8, 288) = 5792.35	0.994	[0.007–0.058]
Leachate concentration	F(4, 288) = 9521.55	0.992	[0.007–0.058]
Time × Cement type	F(40, 288) = 1046.82	0.993	[0.077–0.176]
Time × Concentration	F(20, 288) = 886.62	0.984	[0.032–0.109]
Cement type × Concentration	F(32, 288) = 426.64	0.979	[0.059–0.151]
Time × Cement × Concentration	F(160, 288) = 139.73	0.987	[0.296–0.421]

**Table 4:** Evaluation of viable, late apoptotic, and necrotic hDPSCs apoptosis/necrosis analysis results among different groups after 24 h of incubation with freshly mixed cement 50% leachates.

Group	Viable Cells	Late Apoptotic Cells	Necrotic Cells
Negative control	86.46 ± 6.07 ^A^	6.87 ± 2.69 ^A^	6.68 ± 3.75 ^A^
Positive control	4.47 ± 1.32 ^B^	89.24 ± 1.8 ^B^	6.29 ± 0.49 ^A^
ProRoot^®^ MTA	84.77 ± 2.15 ^A^	8.93 ± 1.03 ^A^	6.30 ± 1.51 ^A^
MTA Flow ^TM^	88.15 ± 0.79 ^A^	5.29 ± 0.41 ^A^	6.56 ± 0.87 ^A^
MTA Flow^TM^ White	84.90 ± 1.21 ^A^	9.55 ± 0.63 ^A^	5.56 ± 0.69 ^A^

The results are presented as means ± SD. Groups marked with the same superscript letter A or B in the same column do not differ significantly (two way ANOVA test; *p* > 0.05).

## Data Availability

The data presented in this study are available on request from the corresponding author. The data are not publicly available due to privacy and ethical restrictions related to laboratory protocols and institutional policy.

## Supplementary Materials

The following supporting information can be downloaded at: https://www.mdpi.com/article/10.3390/jfb16070252/s1, Table S1: Pairwise comparison analysis of hDPSCs proliferation of hydraulic calcium silicate cement type for hDPSCs proliferation at 100% concentration and all time points; Table S2: Pairwise comparison analysis of hDPSCs proliferation of hydraulic calcium silicate cement type for hDPSCs proliferation at 50% concentration and all time points; Table S3: Pairwise comparison analysis of hDPSCs proliferation of hydraulic calcium silicate cement type for hDPSCs proliferation at 25% concentration and all time points; Table S4: Pairwise comparison analysis of hDPSCs proliferation of hydraulic calcium silicate cement type for hDPSCs proliferation at 12.5% concentration and all time points.

## Abbreviations

The following abbreviations are used in this manuscript:

hDPSCs	Human dental pulp stem cells
MTA	Mineral trioxide aggregate
HCSC	Hydraulic Calcium Silicate-Based cement
NegativeCG	Negative control group
PositveCG	Positive control group
ProRootCG	ProRoot^®^ MTA group
MFWhite	MTA Flow^TM^ White
MF	MTA Flow TM
