# Supplementary material for: Biocompatibility of Hydraulic Calcium Silicate-Based Cement MTA FlowTM on Human Dental Pulp Stem Cells In Vitro
Source: J Funct Biomater. 2025 Jul 7;16(7):252. doi: 10.3390/jfb16070252 (PMC12294829; doi:10.3390/jfb16070252)
Supplement: Supplementary file 1 [file jfb-16-00252-s001.zip › jfb-3705316-supplementary.pdf]

Supplementary material

Table S1. Pairwise comparison analysis of hDPSCs proliferation of hydraulic calcium silicate cement type for hDPSCs proliferation at 100% concentration and all time points.

100% Concentration						
**Multiple comparison group**	**Time point**	**Mean Difference**	**95% CI**	**Adjusted P Value**	**Mean 1**	**Mean 2**
Negative control vs. Positive control	2h	1.093	0.7276 to 1.458	0.0022	1	0.001259
	24h	1.878	1.652 to 2.104	<0.0001	1.878	0.0000005753
	48h	2.701	2.310 to 3.092	<0.0001	2.715	0.01394
	72h	4.683	4.304 to 5.061	<0.0001	4.683	0.0004608
	96h	7.46	7.363 to 7.556	<0.0001	7.468	0.008252
	120h	10.64	10.40 to 10.88	<0.0001	10.65	0.005826
Negative control vs. ProRoot® MTA	2h	1.03	0.6904 to 1.369	0.0011	1	0.06456
	24h	1.823	1.621 to 2.024	<0.0001	1.878	0.05561
	48h	2.653	2.276 to 3.031	<0.0001	2.715	0.06171
	72h	4.623	4.246 to 5.001	<0.0001	4.683	0.05994
	96h	7.452	7.362 to 7.542	<0.0001	7.468	0.0163
	120h	10.59	10.38 to 10.81	<0.0001	10.65	0.05565
Negative control vs. MTA Flow™ White	2h	0.5128	0.1882 to 0.8373	0.008	1	0.5814
	24h	1.161	0.9456 to 1.377	<0.0001	1.878	0.717
	48h	1.993	1.644 to 2.343	<0.0001	2.715	0.7218
	72h	3.94	3.603 to 4.277	<0.0001	4.683	0.7432
	96h	6.836	6.695 to 6.976	<0.0001	7.468	0.6324
	120h	10.11	9.765 to 10.45	<0.0001	10.65	0.5403
Negative control vs. MTA Flow™	2h	0.7844	0.4597 to 1.109	0.0013	1	0.3097
	24h	1.08	0.8665 to 1.293	0.0003	1.878	0.7988
	48h	1.755	1.407 to 2.102	<0.0001	2.715	0.9605
	72h	3.808	3.461 to 4.156	<0.0001	4.683	0.8749
	96h	6.462	6.047 to 6.878	<0.0001	7.468	1.005
	120h	9.754	9.541 to 9.968	<0.0001	10.65	0.893
Positive control vs. ProRoot® MTA	2h	-0.0633	-0.1841 to 0.05751	0.2382	0.001259	0.06456
	24h	-0.05561	-0.1716 to 0.06040	0.283	0.0000005753	0.05561
	48h	-0.04777	-0.1281 to 0.03258	0.1839	0.01394	0.06171
	72h	-0.05948	-0.08194 to -0.03701	0.0031	0.0004608	0.05994
	96h	-0.008051	-0.04905 to 0.03295	0.9	0.008252	0.0163
	120h	-0.04983	-0.1808 to 0.08119	0.4264	0.005826	0.05565
Positive control vs. MTA Flow™ White	2h	-0.5801	-0.7930 to -0.3672	0.0028	0.001259	0.5814
	24h	-0.717	-0.9203 to -0.5137	0.0014	0.0000005753	0.717
	48h	-0.7079	-0.8993 to -0.5164	0.0013	0.01394	0.7218
	72h	-0.7428	-0.9417 to -0.5438	0.0013	0.0004608	0.7432
	96h	-0.6241	-0.7798 to -0.4684	0.0009	0.008252	0.6324
	120h	-0.5345	-0.9189 to -0.1501	0.0201	0.005826	0.5403
Positive control vs. MTA Flow™	2h	-0.3084	-0.5120 to -0.1049	0.0157	0.001259	0.3097
	24h	-0.7988	-0.8625 to -0.7350	<0.0001	0.0000005753	0.7988
	48h	-0.9465	-1.174 to -0.7192	0.001	0.01394	0.9605
	72h	-0.8745	-1.014 to -0.7347	0.0002	0.0004608	0.8749
	96h	-0.9972	-1.430 to -0.5647	0.0046	0.008252	1.005
	120h	-0.8872	-1.029 to -0.7450	0.0002	0.005826	0.893
ProRoot® MTA vs. MTA Flow™ White	2h	-0.5168	-0.7061 to -0.3275	0.0007	0.06456	0.5814
	24h	-0.6614	-0.8420 to -0.4808	0.0002	0.05561	0.717
	48h	-0.6601	-0.8337 to -0.4865	0.0003	0.06171	0.7218
	72h	-0.6833	-0.8800 to -0.4866	0.0014	0.05994	0.7432
	96h	-0.6161	-0.7647 to -0.4674	0.0005	0.0163	0.6324
	120h	-0.4847	-0.8405 to -0.1289	0.0184	0.05565	0.5403
ProRoot® MTA vs. MTA Flow™	2h	-0.2451	-0.4261 to -0.06416	0.0151	0.06456	0.3097
	24h	-0.7432	-0.8463 to -0.6400	<0.0001	0.05561	0.7988
	48h	-0.8988	-1.108 to -0.6891	0.0003	0.06171	0.9605
	72h	-0.815	-0.9517 to -0.6783	0.0002	0.05994	0.8749
	96h	-0.9892	-1.418 to -0.5600	0.0045	0.0163	1.005
	120h	-0.8373	-0.9748 to -0.6999	<0.0001	0.05565	0.893

MTA Flow™ White vs. MTA Flow™	2h	0.2717	0.06305 to 0.4803	0.0155	0.5814	0.3097
	24h	-0.08177	-0.2715 to 0.1080	0.3986	0.717	0.7988
	48h	-0.2387	-0.4512 to -0.02618	0.0307	0.7218	0.9605
	72h	-0.1317	-0.3104 to 0.04700	0.1465	0.7432	0.8749
	96h	-0.3731	-0.7715 to 0.02525	0.0613	0.6324	1.005
	120h	-0.3526	-0.7055 to 0.0002171	0.0501	0.5403	0.893

Data are presented as mean difference, confidence intervals, adjusted P value and means. Note: Bonferroni adjustments were made to the level at which statistical significance was declared by dividing the current level of statistical significance at ($p<0.05$) by the number of simple-simple main effects analysed. Therefore, a simple-simple main effect was declared statistically significant when $p<0.0.017$ ($p<0.05/3$).

Table S2. Pairwise comparison analysis of hDPSCs proliferation of hydraulic calcium silicate cement type for hDPSCs proliferation at 50% concentration and all time points.

**50% Concentration**						
**Multiple comparison group**	**Time point**	**Mean Difference**	**95% CI**	**Adjusted P Value**	**Mean 1**	**Mean 2**
Negative control vs. Positive control	2h	0.9155	0.5508 to 1.280	0.0036	1	0.1786
	24h	1.813	1.610 to 2.015	<0.0001	1.878	0.06566
	48h	2.376	2.028 to 2.723	<0.0001	2.715	0.3393
	72h	4.517	4.163 to 4.872	<0.0001	4.683	0.1658
	96h	7.437	7.344 to 7.529	<0.0001	7.468	0.03129
	120h	10.46	10.19 to 10.73	<0.0001	10.65	0.1867
Negative control vs. ProRoot® MTA	2h	0.3068	-0.01850 to 0.6321	0.0622	1	0.7873
	24h	0.3688	0.06838 to 0.6693	0.0218	1.878	1.509
	48h	0.6541	0.2413 to 1.067	0.0059	2.715	2.061
	72h	1.676	1.338 to 2.014	<0.0001	4.683	3.007
	96h	4.019	3.768 to 4.271	<0.0001	7.468	3.449
	120h	6.041	5.673 to 6.409	<0.0001	10.65	4.606
Negative control vs. MTA Flow™ White	2h	0.1758	-0.1658 to 0.5174	0.3881	1	0.9183
	24h	0.2766	0.06842 to 0.4847	0.0145	1.878	1.602
	48h	0.8578	0.5023 to 1.213	0.0021	2.715	1.857
	72h	2.037	1.701 to 2.374	<0.0001	4.683	2.646
	96h	4.129	3.775 to 4.483	<0.0001	7.468	3.339
	120h	6.031	5.626 to 6.436	<0.0001	10.65	4.616
Negative control vs. MTA Flow™	2h	0.6429	0.3182 to 0.9676	0.0031	1	0.4512
	24h	0.4106	0.2098 to 0.6113	0.0027	1.878	1.468
	48h	0.5732	0.2212 to 0.9252	0.0086	2.715	2.142
	72h	1.949	1.575 to 2.323	0.0004	4.683	2.734
	96h	4.148	3.807 to 4.488	<0.0001	7.468	3.32
	120h	6.236	5.970 to 6.502	<0.0001	10.65	4.411
Positive control vs. ProRoot® MTA	2h	-0.6087	-0.8409 to -0.3766	0.0031	0.1786	0.7873
	24h	-1.444	-1.756 to -1.131	0.0003	0.06566	1.509
	48h	-1.722	-2.106 to -1.337	0.0001	0.3393	2.061
	72h	-2.841	-3.065 to -2.618	<0.0001	0.1658	3.007
	96h	-3.418	-3.674 to -3.161	<0.0001	0.03129	3.449
	120h	-4.42	-4.792 to -4.047	<0.0001	0.1867	4.606
Positive control vs. MTA Flow™ White	2h	-0.7397	-1.048 to -0.4312	0.0041	0.1786	0.9183
	24h	-1.536	-1.697 to -1.375	<0.0001	0.06566	1.602
	48h	-1.518	-1.716 to -1.320	<0.0001	0.3393	1.857
	72h	-2.48	-2.663 to -2.297	<0.0001	0.1658	2.646
	96h	-3.308	-3.667 to -2.948	<0.0001	0.03129	3.339
	120h	-4.43	-4.835 to -4.024	<0.0001	0.1867	4.616
Positive control vs. MTA Flow™	2h	-0.2727	-0.4748 to -0.07054	0.0218	0.1786	0.4512
	24h	-1.402	-1.521 to -1.283	<0.0001	0.06566	1.468
	48h	-1.802	-2.004 to -1.600	<0.0001	0.3393	2.142
	72h	-2.568	-2.672 to -2.464	<0.0001	0.1658	2.734
	96h	-3.289	-3.635 to -2.943	<0.0001	0.03129	3.32
	120h	-4.225	-4.515 to -3.935	<0.0001	0.1867	4.411
ProRoot® MTA vs.	2h	-0.131	-0.4119 to 0.1499	0.4569	0.7873	0.9183
	24h	-0.09227	-0.3913 to 0.2067	0.7139	1.509	1.602
	48h	0.2037	-0.1920 to 0.5995	0.2964	2.061	1.857

MTA Flow™ White	72h	0.3615	0.1307 to 0.5924	0.0065	3.007	2.646
	96h	0.1099	-0.2279 to 0.4478	0.7236	3.449	3.339
	120h	-0.01002	-0.4464 to 0.4264	>0.9999	4.606	4.616
ProRoot® MTA vs. MTA Flow™	2h	0.3361	0.1160 to 0.5561	0.0072	0.7873	0.4512
	24h	0.04173	-0.2664 to 0.3498	0.9644	1.509	1.468
	48h	-0.08092	-0.4725 to 0.3106	0.8753	2.061	2.142
	72h	0.2734	0.03180 to 0.5149	0.0352	3.007	2.734
	96h	0.1288	-0.1992 to 0.4568	0.5943	3.449	3.32
	120h	0.195	-0.1762 to 0.5661	0.3537	4.606	4.411
MTA Flow™ White vs. MTA Flow™	2h	0.4671	0.1912 to 0.7430	0.0053	0.9183	0.4512
	24h	0.134	-0.02871 to 0.2967	0.1017	1.602	1.468
	48h	-0.2847	-0.4523 to -0.1170	0.0042	1.857	2.142
	72h	-0.08818	-0.2862 to 0.1098	0.3512	2.646	2.734
	96h	0.01889	-0.3486 to 0.3864	0.9996	3.339	3.32
	120h	0.205	-0.1997 to 0.6097	0.3699	4.616	4.411

Data are presented as mean difference, confidence intervals, adjusted P value and means. Note: Bonferroni adjustments were made to the level at which statistical significance was declared by dividing the current level of statistical significance at ($p<0.05$) by the number of simple-simple main effects analysed. Therefore, a simple-simple main effect was declared statistically significant when p<0.0.017 ($p<0.05/3$).

Table S3. Pairwise comparison analysis of hDPSCs proliferation of hydraulic calcium silicate cement type for hDPSCs proliferation at 25% concentration and all time points.

**25% Concentration**						
**Multiple comparison group**	**Time point**	**Mean Difference**	**95% CI**	**Adjusted P Value**	**Mean 1**	**Mean 2**
Negative control vs. Positive control	2h	0.6291	0.3032 to 0.9549	0.0039	1	0.465
	24h	0.877	0.6688 to 1.085	<0.0001	1.878	1.001
	48h	1.055	0.6876 to 1.423	0.0016	2.715	1.66
	72h	2.291	1.908 to 2.673	<0.0001	4.683	2.393
	96h	4.059	3.817 to 4.300	<0.0001	7.468	3.409
	120h	6.847	6.633 to 7.061	<0.0001	10.65	3.8
Negative control vs. ProRoot® MTA	2h	0.2962	-0.03237 to 0.6248	0.0704	1	0.7979
	24h	0.4084	0.1992 to 0.6176	0.0021	1.878	1.47
	48h	0.1997	-0.1717 to 0.5711	0.2485	2.715	2.515
	72h	1.128	0.7778 to 1.477	0.0008	4.683	3.555
	96h	1.751	1.586 to 1.917	<0.0001	7.468	5.717
	120h	3.121	2.807 to 3.435	<0.0001	10.65	7.527
Negative control vs. MTA Flow™ White	2h	0.1484	-0.1762 to 0.4729	0.4388	1	0.9458
	24h	0.1996	-0.001098 to 0.4004	0.051	1.878	1.679
	48h	0.3868	0.006781 to 0.7668	0.0465	2.715	2.328
	72h	1.093	0.7547 to 1.431	0.0004	4.683	3.59
	96h	1.682	1.314 to 2.050	0.0004	7.468	5.786
	120h	2.682	2.416 to 2.948	<0.0001	10.65	7.965
Negative control vs. MTA Flow™	2h	0.1484	-0.1762 to 0.4729	0.4388	1	0.5735
	24h	0.3825	0.1812 to 0.5838	0.004	1.878	1.496
	48h	0.2114	-0.1449 to 0.5677	0.2756	2.715	2.504
	72h	1	0.6456 to 1.355	0.0003	4.683	3.683
	96h	1.662	1.332 to 1.993	0.0003	7.468	5.806
	120h	3.172	2.953 to 3.390	<0.0001	10.65	7.476
Positive control vs. ProRoot® MTA	2h	-0.3329	-0.5103 to -0.1554	0.0025	0.465	0.7979
	24h	-0.4686	-0.6520 to -0.2852	0.0004	1.001	1.47
	48h	-0.8557	-0.9633 to -0.7482	<0.0001	1.66	2.515
	72h	-1.163	-1.521 to -0.8050	0.0008	2.393	3.555
	96h	-2.307	-2.544 to -2.071	<0.0001	3.409	5.717
	120h	-3.726	-4.043 to -3.410	<0.0001	3.8	7.527
Positive control vs. MTA Flow™ White	2h	-0.4807	-0.6829 to -0.2785	0.0007	0.465	0.9458
	24h	-0.6773	-0.8422 to -0.5125	<0.0001	1.001	1.679
	48h	-0.6686	-1.012 to -0.3248	0.0058	1.66	2.328
	72h	-1.198	-1.543 to -0.8521	0.0003	2.393	3.59
	96h	-2.377	-2.724 to -2.030	<0.0001	3.409	5.786
	120h	-4.165	-4.419 to -3.911	<0.0001	3.8	7.965

Positive control vs. MTA Flow™	2h	-0.1084	-0.3056 to 0.08876	0.3397	0.465	0.5735
	24h	-0.4945	-0.6564 to -0.3326	0.0003	1.001	1.496
	48h	-0.844	-1.119 to -0.5695	0.0009	1.66	2.504
	72h	-1.29	-1.650 to -0.9302	<0.0001	2.393	3.683
	96h	-2.396	-2.715 to -2.078	<0.0001	3.409	5.806
	120h	-3.676	-3.844 to -3.507	<0.0001	3.8	7.476
ProRoot® MTA vs. MTA Flow™ White	2h	-0.1479	-0.3436 to 0.04789	0.1384	0.7979	0.9458
	24h	-0.2087	-0.3760 to -0.04146	0.0198	1.47	1.679
	48h	0.1871	-0.1604 to 0.5345	0.2506	2.515	2.328
	72h	-0.03485	-0.2052 to 0.1355	0.9206	3.555	3.59
	96h	-0.06952	-0.4165 to 0.2775	0.8997	5.717	5.786
	120h	-0.4389	-0.7629 to -0.1149	0.0132	7.527	7.965
ProRoot® MTA vs. MTA Flow™	2h	0.2244	0.03442 to 0.4144	0.0247	0.7979	0.5735
	24h	-0.0259	-0.1905 to 0.1388	0.9642	1.47	1.496
	48h	0.01168	-0.2661 to 0.2894	0.9995	2.515	2.504
	72h	-0.1272	-0.4203 to 0.1659	0.4238	3.555	3.683
	96h	-0.08877	-0.4011 to 0.2236	0.7609	5.717	5.806
	120h	0.05074	-0.2617 to 0.3632	0.9514	7.527	7.476
MTA Flow™ White vs. MTA Flow™	2h	0.3723	0.1620 to 0.5826	0.0033	0.9458	0.5735
	24h	0.1828	0.05476 to 0.3109	0.0101	1.679	1.496
	48h	-0.1754	-0.5154 to 0.1646	0.3836	2.328	2.504
	72h	-0.09236	-0.3789 to 0.1942	0.6996	3.59	3.683
	96h	-0.01925	-0.3902 to 0.3517	0.9996	5.786	5.806
	120h	0.4896	0.2358 to 0.7435	0.003	7.965	7.476

Data are presented as mean difference, confidence intervals, adjusted P value and means. Note: Bonferroni adjustments were made to the level at which statistical significance was declared by dividing the current level of statistical significance at (p<0.05) by the number of simple-simple main effects analysed. Therefore, a simple-simple main effect was declared statistically significant when p<0.0.017 (p<0.05/3).

Table S4. Pairwise comparison analysis of hDPSCs proliferation of hydraulic calcium silicate cement type for hDPSCs proliferation at 12.5% concentration and all time points.

12.5% Concentration						
**Multiple comparison group**	**Time point**	**Mean Difference**	**95% CI**	**Adjusted P Value**	**Mean 1**	**Mean 2**
Negative control vs. Positive control	2h	0.6428	0.3178 to 0.9677	0.0033	1	0.4514
	24h	0.5419	0.2614 to 0.8224	0.0025	1.878	1.336
	48h	0.8401	0.4921 to 1.188	0.001	2.715	1.875
	72h	1.502	1.164 to 1.840	0.0001	4.683	3.181
	96h	1.915	1.387 to 2.443	0.001	7.468	5.553
	120h	3.203	2.984 to 3.423	<0.0001	10.65	7.444
Negative control vs. ProRoot® MTA	2h	0.3081	-0.02817 to 0.6443	0.0656	1	0.7861
	24h	0.3638	0.08125 to 0.6464	0.0173	1.878	1.514
	48h	0.5474	0.2002 to 0.8947	0.0082	2.715	2.168
	72h	0.7423	0.3160 to 1.169	0.0038	4.683	3.941
	96h	0.4605	0.06091 to 0.8602	0.0329	7.468	7.007
	120h	1.194	0.9775 to 1.410	<0.0001	10.65	9.454
Negative control vs. MTA Flow™ White	2h	0.04696	-0.2828 to 0.3767	0.9632	1	1.047
	24h	0.2237	0.007507 to 0.4400	0.0453	1.878	1.655
	48h	0.463	0.1114 to 0.8145	0.0158	2.715	2.252
	72h	0.5091	0.1728 to 0.8455	0.0094	4.683	4.174
	96h	0.541	0.4498 to 0.6322	<0.0001	7.468	6.927
	120h	1.039	0.8221 to 1.257	<0.0001	10.65	9.608
Negative control vs. MTA Flow™	2h	0.3791	0.05419 to 0.7040	0.0282	1	0.715
	24h	0.2733	0.06801 to 0.4785	0.0147	1.878	1.605
	48h	0.1951	-0.1526 to 0.5427	0.295	2.715	2.52
	72h	0.4905	0.1492 to 0.8319	0.0105	4.683	4.193
	96h	0.3918	0.2458 to 0.5378	0.0007	7.468	7.076
	120h	1.19	0.9754 to 1.404	<0.0001	10.65	9.458
Positive control vs. ProRoot® MTA	2h	-0.3347	-0.5110 to -0.1584	0.003	0.4514	0.7861
	24h	-0.1781	-0.4903 to 0.1341	0.3138	1.336	1.514
	48h	-0.2927	-0.5301 to -0.05519	0.0201	1.875	2.168

	72h	-0.7597	-1.177 to -0.3427	0.0059	3.181	3.941
	96h	-1.454	-1.950 to -0.9585	0.0003	5.553	7.007
	120h	-2.01	-2.188 to -1.832	<0.0001	7.444	9.454
Positive control vs. MTA Flow™ White	2h	-0.5958	-0.7779 to -0.4136	0.0001	0.4514	1.047
	24h	-0.3182	-0.6212 to -0.01509	0.0436	1.336	1.655
	48h	-0.3771	-0.6415 to -0.1128	0.01	1.875	2.252
	72h	-0.9929	-1.203 to -0.7825	<0.0001	3.181	4.174
	96h	-1.374	-1.908 to -0.8397	0.0031	5.553	6.927
	120h	-2.164	-2.344 to -1.984	<0.0001	7.444	9.608
Positive control vs. MTA Flow™	2h	-0.2637	-0.4619 to -0.06544	0.0139	0.4514	0.715
	24h	-0.2687	-0.5449 to 0.007601	0.0552	1.336	1.605
	48h	-0.645	-0.8906 to -0.3994	0.0004	1.875	2.52
	72h	-1.011	-1.257 to -0.7664	<0.0001	3.181	4.193
	96h	-1.523	-2.032 to -1.014	0.0013	5.553	7.076
	120h	-2.014	-2.186 to -1.841	<0.0001	7.444	9.458
ProRoot® MTA vs. MTA Flow™ White	2h	-0.2611	-0.4092 to -0.1130	0.0036	0.7861	1.047
	24h	-0.1401	-0.4460 to 0.1658	0.3236	1.514	1.655
	48h	-0.08448	-0.3402 to 0.1712	0.7236	2.168	2.252
	72h	-0.2332	-0.6442 to 0.1777	0.2636	3.941	4.174
	96h	0.08046	-0.3260 to 0.4869	0.8415	7.007	6.927
	120h	-0.1544	-0.3273 to 0.01852	0.0783	9.454	9.608
ProRoot® MTA vs. MTA Flow™	2h	0.07103	-0.1066 to 0.2486	0.562	0.7861	0.715
	24h	-0.09059	-0.3694 to 0.1882	0.6814	1.514	1.605
	48h	-0.3524	-0.5862 to -0.1186	0.0076	2.168	2.52
	72h	-0.2518	-0.6601 to 0.1565	0.2308	3.941	4.193
	96h	-0.06871	-0.4501 to 0.3126	0.9143	7.007	7.076
	120h	-0.003958	-0.1674 to 0.1595	>0.9999	9.454	9.458
MTA Flow™ White vs. MTA Flow™	2h	0.3321	0.1489 to 0.5154	0.0032	1.047	0.715
	24h	0.04951	-0.1097 to 0.2087	0.6283	1.655	1.605
	48h	-0.2679	-0.5298 to -0.005976	0.0456	2.252	2.52
	72h	-0.01858	-0.2720 to 0.2348	0.9983	4.174	4.193
	96h	-0.1492	-0.2966 to -0.001718	0.0481	6.927	7.076
	120h	0.1504	-0.01614 to 0.3170	0.0748	9.608	9.458

Data are presented as mean difference, confidence intervals, adjusted P value and means. Note: Bonferroni adjustments were made to the level at which statistical significance was declared by dividing the current level of statistical significance at (p<0.05) by the number of simple-simple main effects analysed. Therefore, a simple-simple main effect was declared statistically significant when p<0.0.017 (p<0.05/3).
